# The associations of genetic polymorphisms in *CYP1A2* and *CYP3A4* with clinical outcomes of breast cancer patients in northern China

**DOI:** 10.18632/oncotarget.16359

**Published:** 2017-03-18

**Authors:** Xianan Bai, Jingjing Xie, Shanshan Sun, Xianyu Zhang, Yongdong Jiang, Da Pang

**Affiliations:** ^1^ Department of Breast Surgery, Harbin Medical University Cancer Hospital, Harbin150040, China

**Keywords:** SNP, CYP1A2, CYP3A4, breast cancer, prognosis indicator

## Abstract

**Background:**

*Cytochrome P450 (CYP) 1A2* and *CYP3A4* may play a role in the differentiation of clinical outcomes among breast cancer women. This study aimed to analyze the association of genetic polymorphisms in the *CYP1A2* and *CYP3A4* genes with clinicopathological features, protein expression and prognosis of breast cancer in the northern Chinese population.

**Results:**

Firstly, SNP rs11636419, rs17861162 and rs2470890 in the *CYP1A2* were significantly associated with age and menstruation status. And SNP rs11636419 and rs17861162 were associated with the P53 status. Secondly, SNP rs2470890 was correlated with *CYP1A2* protein expression under the co-dominant and dominant model (*P* = 0.017, *P* = 0.006, respectively). Thirdly, for SNP rs2470890, the Kaplan–Meier 5 year survival curves showed that patients carrying genotypes CT or TT had a worse OS compared with the genotype CC carriers under both codominant and dominant model (*P* < 0.001, *P* < 0.001, respectively).

**Materials and Methods:**

Four single nucleotide polymorphisms (SNPs) were successfully genotyped in 459 breast cancer patients using the SNaPshot method. The associations of four polymorphisms with protein expression and clinicopathological characteristics were evaluated by Pearson's chi-square test. The Cox hazard regression analysis and Kaplan–Meier survival analysis were performed to evaluate the relationship between the SNPs and overall survival (OS) of breast cancer.

**Conclusions:**

*CYP1A2* rs2470890 was significantly associated with the prognosis of patients with breast cancer and could serve as an independent impact factor of prognosis of breast carcinoma.

## INTRODUCTION

Breast cancer is the most common malignancy among women worldwide [1]. The crude mortality of breast cancer doubled from year 1973–1975 to year 2004–2005 in China [2]. It is well known that breast cancer is a multifactorial disease, and that besides sex hormone, environment and lifestyle, the genetic background contributes to promote the development of breast cancer. Although a huge amount of studies reported the involvement of genetic polymorphisms in breast cancer susceptibility, studies of the genetic influence on disease progression and outcome are less frequent [3]. SNPs associated with increased severity or worsening progression of breast cancer would potentially afford a better individualized treatment of patients.

Prolonged exposure to estrogens and their oxidative metabolites is considered a crucial factor for the development and evolution of breast cancer [4]. *CYP1A2* and *CYP3A4*, crucial enzymes belong to *CYP450* superfamily, are responsible for the oxidative metabolism of estradiol and estrone in the adult human liver in addition to the metabolic deactivation of exogenous compounds, including environmental procarcinogens [5]. There are considerable interindividual variations in the expression and activity of *CYP1A2* and *CYP3A4* due to the genetic polymorphisms [6–9], which may be associated with the carcinogenic process [10]. Genetic polymorphisms of *CYP1A2* have been identified as leading to interindividual variation in the susceptibility to a series of cancer, such as cholangiocarcinoma, lung, colorectal and breast cancer [11–15]. In like manner, *CYP3A4* genetic variations have been also reported the functions of increasing predisposition of a wide variety of tumors [16–19]. More importantly, some variations in the *CYP3A4* gene have been observed to play significantly roles in the development and outcome of several types of cancer [20–22]. However, to date, lack of studies involving the effect of single nucleotide polymorphisms (SNPs) in the *CYP1A2* and *CYP3A4* genes on the prognosis and survival of patients with breast cancer.

Thus, we performed a combined analysis of functional significance and Tag SNP strategies to select four potential functional SNPs in the *CYP450* genes *CYP1A2* and *CYP3A4* from the dbSNP and HapMap databases. The minor allele frequency (MAF) of these SNPs was greater than 5%, and the pair-wise *r*^2^ was more than 0.8. As a result, 3 SNPs in *CYP1A2*, including rs11636419 and rs17861162 at the 3′-UTR and rs2470890 at exon 7, and 1 SNP, rs12333983, in *CYP3A4* close to the 3′ site of the gene were identified. In the present study, we carried out this case-only study to evaluate the associations between these four SNPs in the *CYP1A2* and *CYP3A4* genes and clinicopathological characteristics, *CYP1A2* and *CYP3A4* protein expression and prognosis with women from Heilongjiang Province, northern China.

## RESULTS

### Associations between genotypes of the four SNPs in *CYP1A2* and *CYP3A4* and clinicopathological characteristics

The genotype distribution of all of the four SNPs selected for this study did not deviate from Hardy–Weinberg equilibrium (*P* > 0.05). We next analyzed the effects of the four SNPs in the *CYP1A2* and *CYP3A4* genes on a series of clinicopathological parameters in the patient cohort, including age at diagnosis and menstrual status, clinic stage, tumor size, histological grade, LNM and the status of ER, PR, HER2, Ki67 and P53. The clinicopathological features of breast cancer patients are shown in Table 1 and Supplementary Table 1.

**Table 1:** Association between the genotypes and the clinicopathological variables (dominant model)

Characteristics	NO.	rs12333983		*p* value	rs11636419		*p* value	rs17861162		*p* value	rs2470890		*p* value
		TT	TG + GG		AA	AG + GG		CC	CG + GG		CC	CT + TT	
Age (years)	459												
≤ 50		139 (52.5)	126 (47.5)	0.723	146 (55.1)	119 (44.9)	0.025	149 (56.2)	116 (43.8)	0.019	223 (84.2)	42 (15.8)	< 0.001
> 50		105 (54.1)	89 (45.9)		127 (65.5)	67 (34.5)		130 (67.0)	64 (33.0)		135 (69.6)	59 (30.4)	
Menopause status	459												
Pre-menopause		136 (52.1)	125 (47.9)	0.604	139 (53.3)	122 (46.7)	0.002	142 (54.4)	119 (45.6)	0.001	224 (85.8)	37 (14.2)	< 0.001
Post-menopause		108 (54.5)	90 (45.5)		134 (67.7)	64 (32.3)		137 (69.2)	61 (30.8)		134 (67.7)	64 (32.3)	
TNM stage	403												
I, II		194 (52.7)	174 (47.3)	0.859	221 (60.1)	147 (39.9)	0.995	227 (61.7)	141 (38.3)	0.845	289 (78.5)	79 (21.5)	0.840
III, IV		19 (54.3)	16 (45.7)		21(60.0)	14 (40.0)		21 (60.0)	14 (40.0)		28 (80.0)	7 (20.0)	
Tumor stage	396												
T1,T2		195 (52.7)	175 (47.3)	0.622	226 (61.1)	144 (38.9)	0.264	232 (62.7)	138 (37.3)	0.197	290 (78.4)	80 (21.6)	0.774
T3,T4		15 (57.7)	11 (42.3)		13 (50.0)	13 (50.0)		13 (50.0)	13 (50.0)		21 (80.8)	5 (19.2)	
Histological grade	359												
1–2		125 (53.0)	111 (47.0)	0.676	142 (60.2)	94 (39.8)	0.299	145 (61.4)	91 (38.6)	0.406	178 (75.4)	58 (24.6)	0.206
3		68 (55.3)	55 (44.7)		67 (54.5)	56 (45.5)		70 (56.9)	53 (43.1)		100 (81.3)	23 (18.7)	
LNM	446												
Negative		131 (52.6)	118 (47.4)	0.567	152 (61.0)	97 (39.0)	0.568	156 (62.7)	93 (37.3)	0.483	196 (78.7)	53 (21.3)	0.693
Positive		109 (55.3)	88 (44.7)		115 (58.4)	82 (41.6)		117 (59.4)	80 (40.6)		152 (77.2)	45 (22.8)	
ER	422												
Negative		86 (52.8)	77 (47.2)	0.838	93 (57.1)	70 (42.9)	0.297	98 (60.1)	65 (39.9)	0.618	124 (76.1)	39 (23.9)	0.301
Positive		134 (51.7)	125 (48.3)		161 (62.2)	98 (37.8)		162 (62.5)	97 (37.5)		208 (80.3)	51 (19.7)	
PR	422												
Negative		105 (54.1)	89 (45.9)	0.450	113 (58.2)	81 (41.8)	0.452	119 (61.3)	75 (38.7)	0.916	147 (75.8)	47 (24.2)	0.180
Positive		115 (50.4)	113 (49.6)		141 (61.8)	87 (38.2)		141 (61.8)	87 (38.2)		185 (81.1)	43 (18.9)	
Her-2	387												
Negative		186 (51.7)	174 (48.3)	0.257	221 (61.4)	139 (38.6)	0.549	224 (62.2)	136 (37.8)	0.492	286 (79.4)	74 (20.6)	0.266
Positive		17 (63.0)	10 (37.0)		15 (55.6)	12 (44.4)		15 (55.6)	12 (44.4)		19 (70.4)	8 (29.6)	
Ki-67	418												
Negative		74 (48.7)	78 (51.3)	0.283	91 (59.9)	61 (40.1)	0.895	92 (60.5)	60 (39.5)	0.704	120 (78.9)	32 (21.1)	0.928
Positive		144 (54.1)	122 (45.9)		161 (60.5)	105 (39.5)		166 (62.4)	100 (37.6)		209 (78.6)	57 (21.4)	
P53	418												
Negative		168 (51.9)	156 (48.1)	0.819	206 (63.6)	118 (36.4)	0.011	209 (64.5)	115 (35.5)	0.030	261 (80.6)	63 (19.4)	0.087
Positive		50 (53.2)	44 (46.8)		46 (48.9)	48 (51.1)		49 (52.1)	45 (47.9)		68 (72.3)	26 (27.7)	

For *CYP1A2* rs11636419, under the dominant model breast cancer patients with the combined genotype (GG+AG) were more likely to have a younger age, a pre-menopause status and a P53 negative tumor relative to patients with the AA genotype (*P* = 0.025, 0.002 and 0.011, respectively, Table 1). Meanwhile, significant associations were found between the *CYP1A2* rs11636419 genotypes and the status of menstruation and P53 under the codominant model (*P* = 0.008, 0.033, respectively, Supplementary Table 1).

For *CYP1A2* rs17861162, patients with the combined genotype (GG+CG) were more likely to have a younger age, a pre-menopause status and a P53 negative tumor relative to the genotype CC carriers (*P* = 0.019, 0.001 and 0.030, respectively, Table 1). However, only menopause status remained to be significantly associated with the *CYP1A2* rs17861162 genotypes under the codominant model (*P* = 0.006, Supplementary Table 1).

The *CYP1A2* rs2470890 genotypes were significantly correlated with age and menstruation status under both the dominant model and the codominant model (*P* < 0.001, *P* < 0.001; *P* = 0.001, *P* < 0.001, respectively, Table 1 and Supplementary Table 1). The carriers with the combined genotype (TT + TC) were more probably to have an older age and pre-menopausal status compared to patients with the CC genotype.

Unfortunately, no significant associations were found between the *CYP3A4* rs12333983 genotypes and clinical features of patients with breast cancer in our samples under either the dominant model or the codominant model.

### *CYP1A2* and *CYP3A4* protein expression in breast cancer tissues

**Figure 1:**
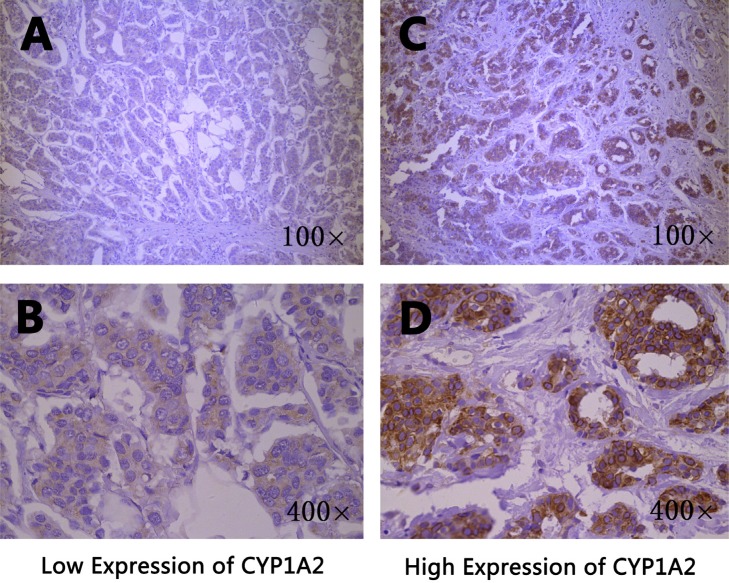
Immunohistochemical staining of *CYP1A2* in breast cancer tissues Staining for each specimen is shown at two magnification: top, 100×; bottom, 400×. *CYP1A2* protein low expression specimens (**A**, **B**); *CYP1A2* protein high expression specimens (**C**, **D**).

**Figure 2:**
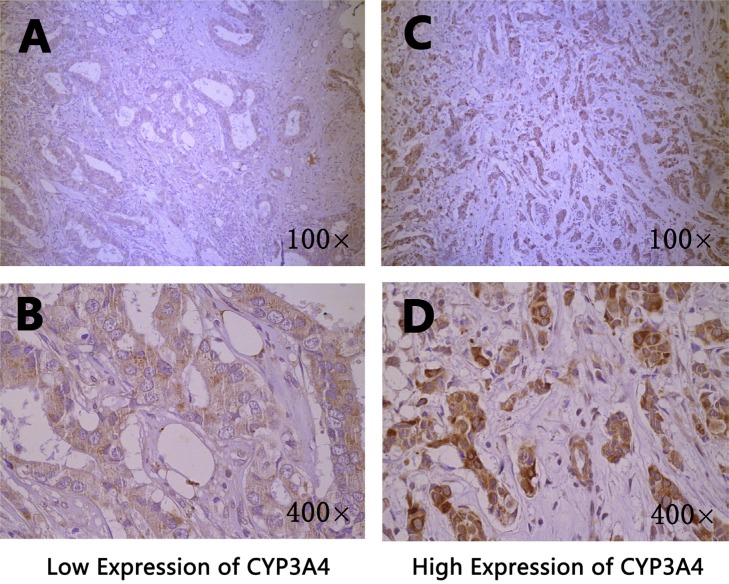
Immunohistochemical staining of *CYP3A4* in breast cancer tissues Staining for each specimen is shown at two magnification: top, 100×; bottom, 400×. *CYP3A4* protein low expression specimens (**A**, **B**); *CYP3A4* protein high expression specimens (**C**, **D**).

The *CYP1A2* and *CYP3A4* protein expression in breast cancer tissues were shown in Figure 1 and Figure 2, respectively. Both *CYP1A2* and *CYP3A4* were predominantly observed staining in the cytoplasm of tumor cells. *CYP1A2* and *CYP3A4* protein expression was shown in 168 breast cancer tissues. Of the 168 breast cancer specimens immunostain with *CYP1A2* protein specific antibody, 117 (69.6 %) were low expression, and 51 (30.4%) were high expression. Of the 168 breast cancer specimens immunostain with *CYP3A4* protein specific antibody, 131 (78.0 %) were low expression, and 37 (22.0%) were high expression. Under the codominant model, we found that SNP rs2470890 was significantly associated with *CYP1A2* protein expression (*P* = 0.017, Table 2). Moreover, the patients with the combined genotypes CT + TT were more likely to have higher *CYP1A2* protein expression when compared to the patients with genotypes CC under the dominant model (*P* = 0.006, Table 2). There were no significant associations between the other three SNPs and *CYP1A2* or *CYP3A4* protein expression either under the codominant or under the dominant model. Additionally, in the present study, there was lack of significant associations between protein expression of *CYP1A2* and *CYP3A4* in breast cancer tissues and OS (Table 3).

**Table 2:** Association of *CYP1A2* and *CYP3A4* genetic polymorphisms with protein expression

SNP	Genotype	NO.	protein expression		*p* value
			Low (%)	High (%)	
rs12333983	TT	86	68 (51.9%)	18 (48.6%)	
	TG	72	56 (42.7%)	16 (43.2%)	
	GG	10	7 (5.3%)	3 (8.1%)	0.806
	TG + GG vs TT	82	63 (48.1%)	19 (51.4%)	0.726
rs11636419	AA	98	65 (55.6%)	33 (64.7%)	
	AG	61	45 (38.5%)	16 (31.4%)	
	GG	9	7 (6.0%)	2 (3.9%)	0.526
	AG + GG vs AA	70	52 (44.4%)	18 (35.3%)	0.269
rs17861162	CC	102	68 (58.1%)	34 (66.7%)	
	CG	56	41 (35.0%)	15 (29.4%)	
	GG	10	8 (6.8%)	2 (3.9%)	0.529
	CG + GG vs CC	66	49 (41.9%)	17 (33.3%)	0.297
rs2470890	CC	120	91 (77.8%)	29 (56.9%)	
	CT	35	20 (17.1%)	15 (29.4%)	
	TT	13	6 (5.1%)	7 (13.7%)	0.017
	CT + TT vs CC	48	26 (22.2%)	22 (43.1%)	0.006

**Table 3:** Prognostic factors in the cox proportional hazards model

Factors	OR	Univariate 95% CI	*p* value	OR	Multivariate 95% CI	*p* value
Age (years)						
≤ 50 / > 50	1.686	(1.188–2.393)	0.003	1.903	(1.193–3.036)	0.007
TNM stage						
I, II / III, IV	2.979	(1.835–4.835)	< 0.001			
Tumor stage						
T1, T2 / T3, T4	2.010	(1.104–3.662)	0.022			
Histological grade						
1–2 / 3	2.051	(1.393–3.019)	< 0.001	1.856	(1.167–2.950)	0.009
Lymph node status						
Negative/Positive	2.907	(1.994–4.237)	< 0.001	3.264	(1.919–5.552)	< 0.001
ER status						
Negative/Positive	0.469	(0.322–0.685)	< 0.001	0.394	(0.249–0.625)	< 0.001
PR status						
Negative/Positive	0.531	(0.362–0.778)	0.001			
Her-2 status						
Negative/Positive	2.005	(1.070–3.758)	0.030			
Ki-67 status						
Negative/Positive	1.323	(0.878–1.993)	0.180			
P53 status						
Negative/Positive	1.190	(0.770–1.839)	0.434			
CYP1A2 Pr-expression						
Low/High	1.016	(0.528–1.955)	0.961			
CYP3A4 Pr-expression						
Low/High	0.798	(0.369–1.723)	0.565			
rs12333983						
AA/GA	0.849	(0.585–1.231)	0.387			
AA/GG	1.237	(0.638–2.400)	0.529			
AA/GA + GG	0.899	(0.633–1.278)	0.553			
rs11636419						
CC/CT	1.108	(0.770–1.595)	0.580			
CC/TT	0.746	(0.302–1.847)	0.527			
CC/CT + TT	1.060	(0.744–1.509)	0.748			
rs17861162						
CC/CG	1.016	(0.699–1.477)	0.932			
CC/GG	1.022	(0.471–2.216)	0.956			
CC/CG + GG	1.017	(0.712–1.453)	0.926			
rs2470890						
CC/CT	1.933	(1.295–2.884)	0.001	1.557	(0.910–2.665)	0.106
CC/TT	3.006	(1.558–5.799)	0.001	3.410	(1.535–7.575)	0.003
CC/CT + TT	2.103	(1.457–3.035)	< 0.001	1.850	(1.148–2.981)	0.012

OR: odds ratio; CI: confidence interval; Pr-expression: protein expression. Note: (A/B), A is the reference.

### Associations between genotypes of the four SNPs in *CYP1A2* and *CYP3A4* and OS

**Figure 3:**
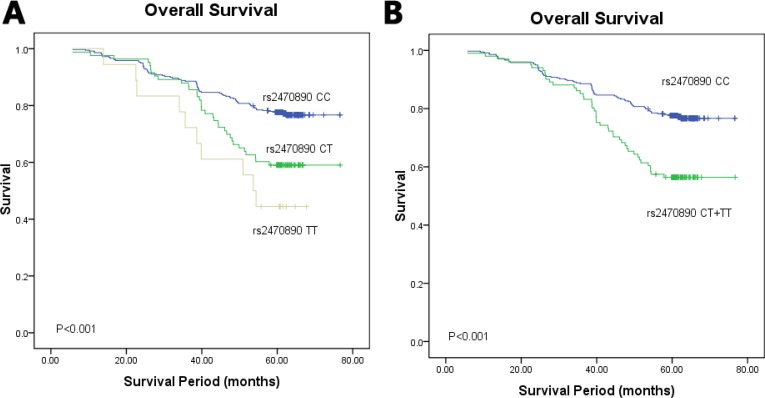
Kaplan–Meier survival curves of breast cancer patients carrying different genotypes of *CYP1A2* rs2470890 (**A**) The codominant model; χ^2^ = 19.150, *P* < 0.001. The mean survival time of CC, CT and TT genotype carriers were 66.967, 95% CI 65.001–68.933; 61.057, 95% CI 56.683–65.431; 50.445, 95% CI 41.994–58.915, respectively; (**B**) the dominant model; χ^2^ = 16.528, *P* < 0.001. The mean survival time of CC, CT + TT genotype carriers were 66.967, 95% CI 65.001–68.933; 59.875, 95% CI 55.812–63.937, respectively.

In stratification analysis for different genotypes of the four SNPs in *CYP1A2* and *CYP3A4*, the Kaplan–Meier survival curve showed a significant association between the *CYP1A2* rs2470890 polymorphism and OS among the 459 study breast cancer patients (*P* < 0.001, Figure 3A). In codominant model, patients with the CC genotype showed significantly improved OS, whereas patients with the TT genotype showed relatively worse OS. Consistent with the above findings, in dominant model patients with the combined genotype (TT+TC) had a worse OS than patients with the CC genotype (*P* < 0.001, Figure 3B). However, the other three SNPs were not associated with OS (data was not shown).

Multivariate analysis with hazard ratio (HR) and corresponding 95% confidence interval (CI) was further performed by adjusting for other variables. The multivariable Cox proportional hazards model showed that in dominant model patients with the *CYP1A2* rs2470890 TT+TC genotypes had significantly worse OS than those with the CC genotype (HR = 1.850, 95% CI 1.148–2.981, *P* = 0.012, Table 3). Meanwhile, breast cancer patients carrying the *CYP1A2* rs2470890 TT genotype had a poorer OS compared to those with the CC genotype (HR = 3.410, 95% CI 1.535–7.575, *P* = 0.003, Table 3). Besides, as we expected, age at diagnosis, histological grade, ER and LNM were associated with worse OS in the multivariable Cox hazard regression analysis (*P* = 0.007, *P* = 0.009, *P* < 0.001 and *P* < 0.001, respectively, Table 3). These data support that *CYP1A2* rs2470890 may be an independent prognostic factor of OS in breast cancer after radical mastectomy.

## DISCUSSION

To the best of our knowledge, we are the first to analyse the association of the four *CYP450* SNPs, including rs11636419, rs17861162, and rs2470890 in the *CYP1A2* gene and rs12333983 in the *CYP3A4* gene, with the clinicopathological features and prognosis of breast cancer patients. In the present study, we found that SNPs rs11636419, rs17861162 and rs2470890 in the *CYP1A2* gene were associated with some clinical features. More importantly, *CYP1A2* rs2470890 allele T was significantly correlated with unfavourable prognosis of breast cancer patients. The data suggested that *CYP1A2* rs2470890 might serve as a novel genetic indicator to evaluate breast cancer prognosis and guide clinical therapy.

*CYP1A2* and *CYP3A4* have key roles in the metabolic activation of pre-carcinogenes and catalyse the metabolism of endogenous substrates such as retinoic and bile acids and steroid hormones such as testosterone and oestrogen [23]. The level of estrogen associated with breast cancer risk of postmenopausal women is directly related to menstruation status and age of the patients [24]. In the present study, we found that SNPs rs11636419, rs17861162 and rs2470890 were significantly associated with age and menstruation status of breast cancer patients. Similarly, coffee consumption combined with CYP1A2*1F genotype was demonstrated to modify age at breast cancer diagnosis and estrogen receptor status [25]. Moreover, a meta-analysis of 46 case–control studies indicated that CYP1A2*1F polymorphism was associated with estrogen-related breast and ovarian cancer risk, but not lung, colorectal, bladder, endometrial, pancreatic and gastric cancer [13]. These indicated that *CYP1A2* genetic polymorphisms might be associated with enzyme inducibility and enzymatic activity, resulting in metabolic disorders of estrogen/progesterone and thereby contributing to increased susceptibility to breast cancer [26]. The results indicated that the three SNPs in the *CYP1A2* gene might alter the activity of *CYP1A2* which act on estrogen metabolism and thus influence the susceptibility of different period women to breast cancer.

*CYP1A2* rs2470890 is a C/T synonymous variation which was first identified in the Russian population [27]. Chen et al. [28] found that the allele C was associated with increased hepatocellular carcinoma (HCC) susceptibility in Chinese population, in HBsAg seronegative individuals, and in heavy smokers. A meta-analysis showed no evidence of significant associations between *CYP1A2* rs2470890 and lung cancer risk among Caucasian and Latinos [14]. However, thus far, there has been no evidence involving the role of *CYP1A2* rs2470890 played in breast cancer. In our study, we found strong correlations of *CYP1A2* rs2470890 with age and menstrual status under the dominant model as well as under the codominant model. Kaplan–Meier 5 year survival curves and multivariate analysis demonstrated breast cancer patients with the *CYP1A2* rs2470890 allele T suffered worse OS compared to wild type allele carriers. Besides, rs2470890 was significantly associated with the *CYP1A2* protein expression not only under the codominant model but also under the dominant model. In the light of the above findings, *CYP1A2* rs2470890 might have an effect on the progression of breast cancer and could serve as a novel prognostic biomarker.

SNPs rs11636419 and rs17861162 are both located at the 3′-UTR of the *CYP1A2* gene. In the previous study, our team found they were associated with the dose of epidural ropivacaine in patients undergoing breast cancer surgery [29]. In the present study, we found the two polymorphisms were significantly correlated with age and the status of menstruation and P53. However, we did not find the evidence concerning their influence on breast cancer survival.

In addition, SNP rs12333983 is an A/T variation in close proximity to the *CYP3A4* gene 3′ end. In our study, we did not find any association of *CYP1A2* rs12333983 with clinicopathological characteristics or prognosis of breast cancer. To date, there are no references to the associations between *CYP1A2* rs12333983 and clinicopathological characteristics and prognosis of any cancer.

In our study, we did not find any association of protein expression of *CYP1A2* and *CYP3A4* in breast cancer tissues with the prognosis of patients. In like manner, no significant correlation between *CYP3A4* expression and clinicopathological factors as well as disease site of breast cancer was observed in other studies [30, 31]. Nevertheless, *CYP1A2* protein expression in noncancerous liver tissue was identified as the predictive candidate for postoperative recurrence of HCC [32]. The different results might due to the entirely different tissues employed in the studies. In any case, the above results need to be further investigated in larger numbers of cohorts and multicenter studies.

In interpreting our results of the current study, some limitations need to be addressed. Firstly, since the population recruited only from northern China, it does not permit extrapolating the results to other ethnic groups as the allele frequency patterns vary greatly between different ethnic groups. Secondly, when we designed the experiment scheme, we did not consider the associations of *CYP1A2* and *CYP3A4* genetic polymorphisms with the response to treatment and chemotherapeutic drug toxicity. Thus, the participants received combination chemotherapy with excessive potential confounding factors limited further analysis. Thirdly, the size of the current study was only a relatively small number in the specific population.

In summary, the present study indicated that *CYP1A2* rs2470890 was associated with breast cancer prognosis among women in northern China. The findings would promise us a functional profiling of the *CYP1A2* gene and understand the biological processes associated with breast cancer formation and progression. *CYP1A2* rs2470890 alone or in combination with other polymorphisms in the oestrogens metabolism related genes might serve as promising prognostic biomarkers of breast cancer. However, more in-depth studies are still needed to perform in different ethnicities in order to validate the associations between genetic polymorphisms in the *CYP1A2* and *CYP3A4* genes and breast cancer to reveal underlying molecular mechanism.

## MATERIALS AND METHODS

### Study subjects

In this study, a total of 459 breast cancer patients were recruited from the Department of Breast Surgery at Harbin Medical University Cancer Hospital from November 2008 until May 2009. The pathological specimens of patients were diagnosed with breast cancer by two pathologists. The participants were excluded from this study if they were genetically related within three generations or previously received neoadjuvant treatment. The Ethical Committee of Harbin Medical University (Harbin Medical University, 268 Xuefu Road, Nangang District, Harbin, China; the protocol number: 2006-Yan-069; the date of approval: March 18, 2006) approved the present study. After providing informed consent, each participant was interviewed to collect detailed information on their demographic characteristics and provided 5 ml of venous blood. The clinical pathological characteristics information of the 459 patients included was obtained from their medical files (Table 4). The age of the patients at diagnosis was 49.47 ± 10.10 years old (ranging from 27 to 91 years old).

**Table 4:** Summary of the clinicopathologic characteristics of the breast cancer patients

Characteristics		No. of cases	Percent (%)
Age (years)	≤ 50	265	57.7
	> 50	194	42.3
Menopause status	Pre-menopause	261	56.9
	Post-menopause	198	43.1
TNM stage	I, II	368	80.2
	III, IV	35	7.6
	Unknown	56	12.2
Tumor stage	T1–T2	370	80.6
	T3–T4	26	5.7
	Unknown	63	13.7
histological grade	1–2	236	51.4
	3	123	26.8
	Unknown	100	21.8
LNM	Negative	249	54.2
	Positive	197	42.9
	Unknown	13	2.8
ER status	Negative	163	35.5
	Positive	259	56.4
	Unknown	37	8.1
PR status	Negative	194	42.3
	Positive	228	49.7
	Unknown	37	8.1
Her-2 status	Negative	360	78.4
	Positive	27	5.9
	Unknown	72	15.7
Ki-67 status	Negative	152	33.1
	Positive	266	58.0
	Unknown	41	8.9
P53 status	Negative	324	70.6
	Positive	94	20.5
	Unknown	41	8.9

All breast cancer patients were tested for the status of oestrogen receptor (ER), progesterone receptor (PR), human epidermal growth factor receptor 2 (HER2), P53 and Ki67, assayed in paraffin-embedded, formation-fixed tissue. Immunohistochemical staining for ER and PR was performed using a conventional detection method and was considered positive if 1% or more of the nuclei in the invasive component of the tumour were stained. Positive staining for Her-2 was defined based on the percentage of tumour cells and the intensity of membrane staining. No staining observed or membrane staining of fewer than 10% of the tumour cells was scored as 0. Faint or barely perceptible incomplete membrane staining detected in more than 10% of the tumour cells was scored as 1+. Weak to moderately complete membrane staining observed in more than 10% of the tumour cells was scored as 2+. Strong complete membrane staining observed in more than 10% of the tumour cells was scored as 3+. Scores of 0 to 1+ were regarded as negative and 3+ were regarded as positive. We selected a Ki67 index of 14% as the optimal cut point for human visual assessment. For P53, positive staining of more than 10% of the tumour cells was defined as positive tumour expression and staining of 10% or fewer of the cells as negative tumour expression.

### Follow-up

Patients were followed regularly for 5 years at the Third Affiliated Hospital of the Harbin Medical University. Clinical records were obtained from the follow-up department of the hospital. All of the patients were followed until death or the study closing date (June 1, 2014). The OS, which measured death from any case, was the assessment used for the prognostic analyses.

### Genomic DNA extraction and genotyping analysis

Genomic DNA was extracted from ethylene diamine tetraacetic acid (EDTA) anti-coagulated whole blood samples using the AxyPrep Blood Genomic DNA Miniprep Kit (Axygen Biotechnology, Tewksbury, MA, USA). The SNaPshot SNP assay was performed to detect dimorphisms of the four *CYP450* SNPs. Data were analysed using the GeneMapper 4.0 Software (Applied Biosystems, Foster City, CA, USA). For quality control purposes, the genotyping was performed without knowledge of the subjects' status. Moreover, 5% of the samples were randomly selected for repeated genotyping by a different technician and the reproducibility was 100%. The average call rate for all of the SNPs was higher than 99%. The four *CYP450* SNPs were rs11636419, rs17861162 and rs2470890 in the *CYP1A2* gene and rs12333983 in the *CYP3A4* gene.

### Immunohistochemical Staining of *CYP1A2* and *CYP3A4*

The formalin-fixed, paraffin-embedded samples were cut into 4μm and stained with H&E for tumor confirmation. The tissue sections were dried at 70°C for 3 h. After deparaffinization and hydration according to the standard procedures, sections were washed in phosphate-buffered saline (PBS; 3 × 3 min). After washing in distilled water, sections were washed in PBS (3 × 5 min) and were then treated with 0.01 mol/L citrate buffer (pH 6.0) and were exposed to heat induced epitope retrieval for 1 min. The washed sections were treated with 3% H_2_O_2_ for 20 min in the dark. The sections were incubated overnight at 4°C with primary antibody *CYP1A2* (1:100 dilution, a recombinant rabbit monoclonal antibody, BOSTER: PB0574) and *CYP3A4* (1:50 dilution, a recombinant rabbit monoclonal antibody, BOSTER: PB1111). After washing in PBS (3 × 5 min), each section was incubated with the secondary antibody (an anti-rabbit antibody, ZSGB-BIO: PV6001) at 37°C for 30 min. After washing in PBS (3 × 5 min), each section was treated with diaminobenzadine (DAB: ZSGB-BIO: ZLI-9018) working solution at room temperature for 3 min and 3 minutes and 30 seconds, respectively, and then washed in distilled water.

### Evaluation of *CYP1A2* and *CYP3A4* protein expression by immunohistochemistry

The immunohistochemical staining of *CYP1A2* and *CYP3A4* were scored by combining the proportion and intensity of positively stained tumor cells. Staining intensity was classified into four groups: level 0 (no staining), level 1 (weak staining = light yellow), level 2 (moderate staining = yellow brown) and level 3 (strong staining = brown). The percentage (0–100%) of the extent of reactivity was scored as follows: 0 (no positive tumour cells), 1 (fewer than 10% positive tumour cells), 2 (10–50% positive tumour cells) and 3 (more than 50% positive tumour cells). Staining index (SI) was calculated as a proportion score × staining intensity score. The final scores ≤ 4 were considered to be low expression, and the remainder were classified as high expression. The slides were examined by pathologists who were blinded to the clinical data.

### Statistical analyses

The genotype frequencies were tested for Hardy–Weinberg equilibrium using the chi-square test. The associations of polymorphisms in the *CYP1A2* and *CYP3A4* genes with the clinicopathological variables, including age, menstruation status, tumor size, histological grade, lymph node metastasis (LNM), TNM pathologic stage and the status of ER, PR, HER-2, Ki-67 and P53 were evaluated by a Pearson's chi-square test. The Cox proportional hazards model was used to estimate the independent prognostic factors for OS. Risk ratios and their 95% confidence intervals were recorded for each marker. The Kaplan–Meier analysis was applied to compare the survival of the patients with different genotypes. The Pearson's chi-square test was used to evaluate the correlations between SNPs of the *CYP1A2* and *CYP3A4* genes and their protein expression. All statistical tests were two-sided, and a *p* value equal to or less than 0.05 was considered statistically significant. Statistical analyses were performed using SPSS for Windows software (version 16.0; SPSS, Chicago, IL, USA).

## SUPPLEMENTARY MATERIALS FIGURES AND TABLES


