# Supplementary material for: The associations of genetic polymorphisms in CYP1A2 and CYP3A4 with clinical outcomes of breast cancer patients in northern China
Source: Oncotarget. 2017 Mar 18;8(24):38367–77. doi: 10.18632/oncotarget.16359 (PMC5503538; doi:10.18632/oncotarget.16359)
Supplement: Supplementary file 1 [file oncotarget-08-38367-s001.pdf]

The associations of genetic polymorphisms in *CYP1A2* and *CYP3A4* with clinical outcomes of breast cancer patients in northern China

Supplementary Materials

Supplementary Table 1: Association between the genotypes and the clinicopathological variables (codominant model)

Characteristics	NO.	rs12333983			*p* value	rs11636419			*p* value	rs17861162			*p* value	rs2470890			*p* value
		TT	GG	TG		AA	GG	AG		CC	GG	CG		CC	TT	CT	
Age (years)	459																
≤ 50		139	110	16	0.770	146	104	15	0.081	149	100	16	0.062	223	35	7	0.001
		(52.5)	(41.5)	(6.0)		(55.1)	(39.2)	(5.7)		(56.2)	(37.7)	(6.0)		(84.2)	(13.2)	(2.6)	
> 50		105	75	14		127	58	9		130	54	10		135	48	11	
		(54.1)	(38.7)	(40.3)		(65.5)	(29.9)	(4.6)		(67.0)	(27.8)	(5.2)		(69.6)	(24.7)	(5.7)	
Menopause status	459																
Pre-menopause		136	108	17	0.861	139	106	16	0.008	142	102	17	0.006	224	32	5	< 0.001
		(52.1)	(41.4)	(6.5)		(53.3)	(40.6)	(6.1)		(54.4)	(39.1)	(6.5)		(85.8)	(12.3)	(1.9)	
Post-menopause		108	77	13		134	56	8		137	52	9		134	51	13	
		(54.5)	(38.9)	(6.6)		(67.7)	(28.3)	(4.0)		(69.2)	(26.3)	(4.5)		(67.7)	(25.8)	(6.6)	
TNM stage	403																
I, II		194	149	25	0.883	221	126	21	0.758	227	119	22	0.972	289	64	15	0.822
		(52.7)	(40.5)	(6.8)		(60.1)	(34.2)	(5.7)		(61.7)	(32.3)	(6.0)		(78.5)	(17.4)	(4.1)	
III, IV		19	13	3		21	13	1		21	12	2		28	5	2	
		(54.3)	(37.1)	(8.6)		(60.0)	(37.1)	(2.9)		(60.0)	(34.3)	(5.7)		(80.0)	(14.3)	(5.7)	
Tumor stage	396																
T1, T2		195	149	26	0.781	226	126	18	0.507	232	118	20	0.434	290	65	15	0.527
		(52.7)	(40.3)	(7.0)		(61.1)	(34.1)	(4.9)		(62.7)	(31.9)	(5.4)		(78.4)	(17.6)	(4.1)	
T3, T4		15	10	1		13	11	2		13	11	2		21	3	2	
		(57.7)	(38.5)	(3.8)		(50.0)	(42.3)	(7.7)		(50.0)	(42.3)	(7.7)		(80.8)	(11.5)	(7.7)	
Histological grade	359																
1–2		125	99	12	0.156	142	82	12	0.446	145	79	12	0.670	178	47	11	0.444
		(53.0)	(41.9)	(5.1)		(60.2)	(34.7)	(5.1)		(61.4)	(33.5)	(5.1)		(75.4)	(19.9)	(4.7)	
3		68	43	12		67	51	5		70	47	6		100	19	4	
		(55.3)	(35.0)	(9.8)		(54.5)	(41.5)	(4.1)		(56.9)	(38.2)	(4.9)		(81.3)	(15.4)	(3.3)	
LNM	446																
Negative		131	100	18	0.806	152	81	16	0.354	156	76	17	0.338	196	42	11	0.578
		(52.6)	(40.2)	(7.2)		(61.0)	(32.5)	(6.4)		(62.7)	(30.5)	(6.8)		(78.7)	(16.9)	(4.4)	
Positive		109	76	12		115	74	8		117	71	9		152	39	6	
		(55.3)	(38.6)	(6.1)		(58.4)	(37.6)	(4.1)		(59.4)	(36.0)	(4.6)		(77.2)	(19.8)	(3.0)	
ER	422																
Negative		86	64	13	0.506	93	59	11	0.324	98	53	12	0.389	124	29	10	0.288
		(52.8)	(39.3)	(8.0)		(57.1)	(36.2)	(6.7)		(60.1)	(32.5)	(7.4)		(76.1)	(17.8)	(6.1)	
Positive		134	111	14		161	88	10		162	86	11		208	43	8	
		(51.7)	(42.9)	(5.4)		(62.2)	(34.0)	(3.9)		(62.5)	(33.2)	(4.2)		(80.3)	(16.6)	(3.1)	
PR	422																
Negative		105	77	12	0.751	113	70	11	0.693	119	63	12	0.825	147	36	11	0.284
		(54.1)	(39.7)	(6.2)		(58.2)	(36.1)	(5.7)		(61.3)	(32.5)	(6.2)		(75.8)	(18.6)	(5.7)	
Positive		115	98	15		141	77	10		141	76	11		185	36	7	
		(50.4)	(43.0)	(6.6)		(61.8)	(33.8)	(4.4)		(61.8)	(33.3)	(4.8)		(81.1)	(15.8)	(3.1)	
Her-2	387																
Negative		186	151	23	0.454	221	124	15	0.252	224	119	17	0.339	286	59	15	0.504
		(51.7)	(41.9)	(6.4)		(61.4)	(34.4)	(4.2)		(62.2)	(33.1)	(4.7)		(79.4)	(16.4)	(4.2)	
Positive		17	8	2		15	9	3		15	9	3		19	6	2	
		(63.0)	(29.6)	(7.4)		(55.6)	(33.3)	(11.1)		(55.6)	(33.3)	(11.1)		(70.4)	(22.2)	(7.4)	
Ki-67	418																
Negative		74	71	7	0.178	91	52	9	0.817	92	50	10	0.756	120	26	6	0.963
		(48.7)	(46.7)	(4.6)		(59.9)	(34.2)	(5.9)		(60.5)	(32.9)	(6.6)		(78.9)	(17.1)	(3.9)	
Positive		144	102	20		161	93	12		166	87	13		209	45	12	
		(54.1)	(38.3)	(7.5)		(60.5)	(35.0)	(4.5)		(62.4)	(32.7)	(4.9)		(78.6)	(16.9)	(4.5)	
P53	418																
Negative		168	138	18	0.308	206	104	14	0.033	209	99	16	0.090	261	51	12	0.207
		(51.9)	(42.6)	(5.6)		(63.6)	(32.1)	(4.3)		(64.5)	(30.6)	(4.9)		(80.6)	(15.7)	(3.7)	
Positive		50	35	9		46	41	7		49	38	7		68	20	6	
		(53.2)	(37.2)	(9.6)		(48.9)	(43.6)	(7.4)		(52.1)	(40.4)	(7.4)		(72.3)	(21.3)	(6.4)	
